# Community engagement: A central feature of NOSM’s socially accountable distributed medical education

**Published:** 2018-03-27

**Authors:** Roger Strasser, John Hogenbirk, Kristen Jacklin, Marion Maar, Geoffrey Hudson, Wayne Warry, Hoi Cheu, Tim Dubé, Dean Carson

**Affiliations:** 1 Northern Ontario School of Medicine, Ontario, Canada; 2 Centre for Rural and Northern Health, Laurentian University, Ontario, Canada; 3 University of Minnesota Medical School, Minnesota, United States; 4 English Department, Laurentian University, Ontario, Canada; 5 McGill University, Quebec, Canada; 6 Charles Darwin University, Northern Territory, Australia

## Abstract

**Background:**

Northern Ontario School of Medicine (NOSM) serves as the Faculty of Medicine of Lakehead and Laurentian Universities, and views the entire geography of Northern Ontario as its campus. This paper explores how community engagement contributes to achieving social accountability in over 90 sites through NOSM’s distinctive model, Distributed Community Engaged Learning (DCEL).

**Methods:**

Studies involving qualitative and quantitative methods contribute to this paper, which draws on administrative data from NOSM and external sources, as well as surveys and interviews of students, graduates and other informants including the joint NOSM-CRaNHR (Centre for Rural and Northern Health Research) tracking and impact studies.

**Results:**

Community engagement contributes throughout the lifecycle stages of preadmission, admission, and undergraduate medical education. High school students from 70 Northern Ontario communities participate in NOSM’s week-long Health Sciences Summer Camps. The MD admissions process involves approximately 128 volunteers assessing written applications and over 100 volunteer interviewers. Thirty-six Indigenous communities host first year students and third-year students learn their core clinical medicine in 15 communities, throughout Northern Ontario. In general, learners and communities report net benefits from participation in NOSM programs.

**Conclusion:**

Community engagement makes a key contribution to the success of NOSM’s socially accountable distributed medical education.

## Introduction

Northern Ontario is geographically vast (>800,000 sq km) with a volatile resource based economy and forty percent of the population living in rural and remote areas where there are diverse communities and cultural groups, most notably Indigenous and Francophone peoples. The health status of people in the region is much worse than the province as a whole, and there is a chronic shortage of physicians and other health professionals.^1-3^ This situation provided the impetus for the establishment of the Northern Ontario School of Medicine (NOSM), which serves as the Faculty of Medicine of Lakehead University in Thunder Bay (population 120,000) and of Laurentian University in Sudbury (population 160,000).^4^ These two universities are over 1,000 km apart and provide teaching, research, and administrative bases for NOSM, which views the entire geography of Northern Ontario as its campus.^5^

NOSM was established with a social accountability mandate focused on improving the health of Northern Ontarians.^6,7^ The World Health Organization (WHO) defines the “Social Accountability of Medical Schools” as “the obligation to direct their education, research, and service activities towards addressing the priority health concerns of the community, region and the nation that they have a mandate to serve.”^8^ Consistent with its social accountability mandate, NOSM developed Distributed Community Engaged Learning (DCEL) as its distinctive model of medical education and health research.

**Figure 1:**
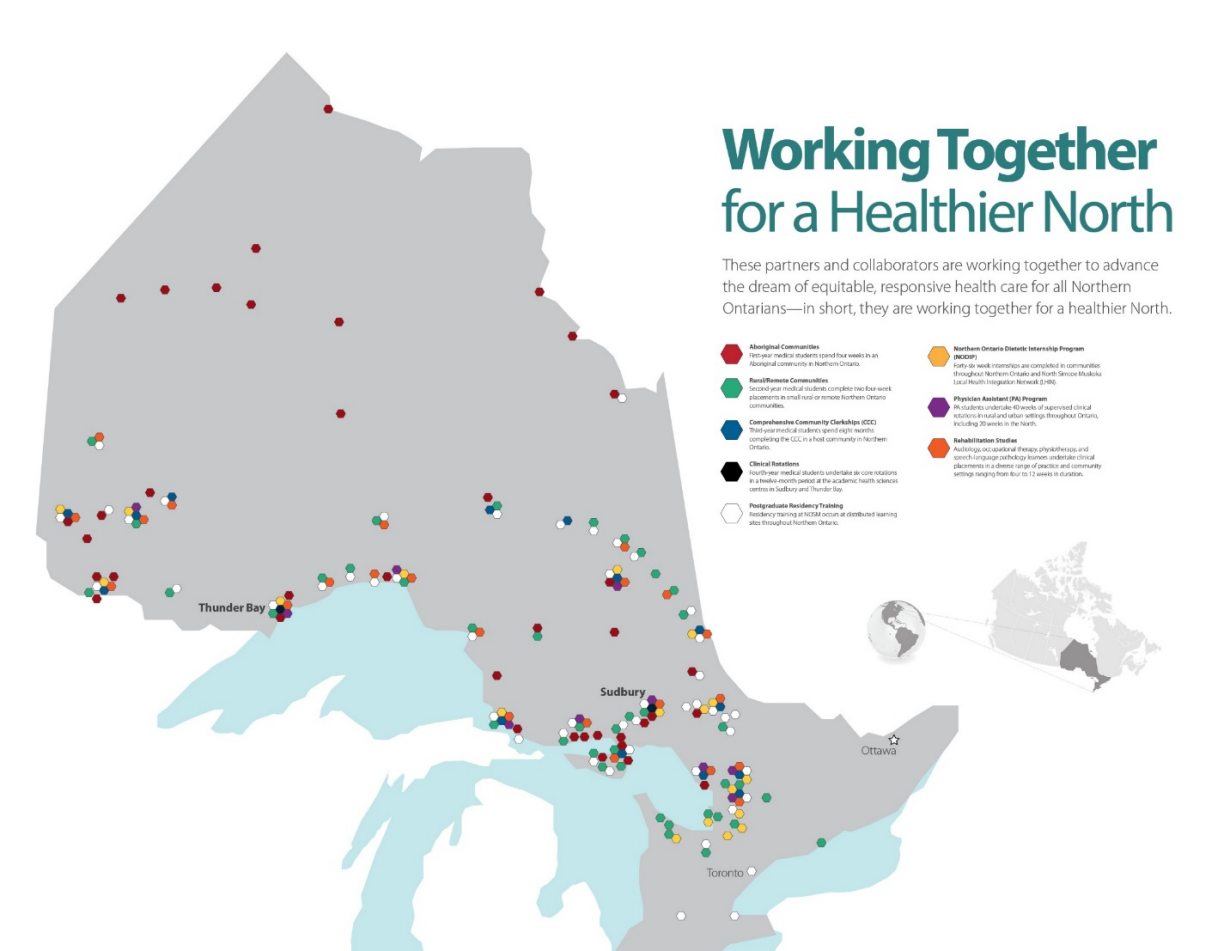
The Northern Ontario School of Medicine’s Distributed Community Engaged Learning in over 90 sites

Distributed learning occurs in over 90 sites (see figure 1) and relies heavily on information and communications technology to connect the sites in real time or asynchronously.^9^ The NOSM Health Sciences Library provides an extensive digital library service which, via the internet, provides access to educational resources and information similar to that in urban teaching hospitals. Community engagement, the central feature of DCEL, occurs through inter-dependent partnerships between the School and the communities for mutual benefit.^10^ In the health system, Community Engagement is a key strategy to fulfill the health system’s commitment to social accountability and transformative change.^11^ Community Engagement in medical education involves active community participation in curriculum development and delivery, as well as hosting learners and helping them to appreciate the social determinants of health at local level.^12^

NOSM has engaged Northern Ontario communities in the development of its academic programs from the outset, starting with a three-day *Getting Started in the North* workshop in January 2003 in preparation for development of the curriculum, attended by 300 participants drawn from communities across the region. Community engagement has continued through regular gatherings involving a wide range of community partners, including members of the local Indigenous and Francophone communities.^12^ Ongoing engagement is demonstrated by the NOSM Strategic Plan 2015-2020, which was developed with input derived from visits touching on 50 communities.^13^ Community members play a vital role in selecting students for the four-year MD program; educating students by serving as standardized patients; and providing local support for students during their community placements.^10^ In their first two years, students experience three Integrated Community Experiences (ICE) placements in rural and Indigenous communities each lasting 4 weeks.^14,15^ During the eight-month Comprehensive Community Clerkship (CCC), third-year students in groups of two to eight live in one of 15 communities, excluding Sudbury and Thunder Bay, to learn core clinical medicine from a family practice and community perspective.^6,9,16^ Much of the CCC curriculum is devised in partnership with the host communities.

NOSM’s research activities are guided by social accountability as well, with a focus on addressing research questions about the health of the people of Northern Ontario and frequently involving community engagement.^17^ An example is the Community Engagement through Research Program, which matches Indigenous communities and medical students with compatible research interests under faculty supervision.

Studies in many countries have shown that the three factors most strongly associated with entering rural and remote practice are: 1) a rural/remote upbringing; 2) positive clinical and educational experiences in rural settings as part of undergraduate medical education; and 3) targeted training for rural practice at the postgraduate level.^18^ Consequently, NOSM’s education and training activities span the career of a physician in Northern Ontario. This begins with programs that encourage Northern Ontario high school students to see themselves as possible future physicians, to achieve the academic requirements to enter university and medical school in Northern Ontario. “Pipeline” initiatives include medical students visiting schools while on mandatory placement in Indigenous communities; science camps led by NOSM students and faculty, which make the connection between high school studies and health careers, particularly for Indigenous and Francophone high school students; and special poster sessions for high school students at the annual NOSM Northern Health Research Conference.^19^

In addition to undergraduate medical education, NOSM offers postgraduate medical education in family medicine and eight other major general specialties.^20^ Like the undergraduate program, these programs recruit residents from Northern Ontario or similar backgrounds and provide DCEL in a range of community and clinical settings in the region. This residency training is targeted on practicing in Northern Ontario or similar rural/remote areas. Once in practice, NOSM provides continuing education/professional development to support and maintain Northern Ontario physicians.^21^ In addition, graduate studies programs (at the masters and PhD levels) are encouraged with the expectation that these doctors will choose to pursue academic careers with NOSM.

Overall, the DCEL model engages communities throughout the physician lifecycle in Northern Ontario. The purpose of this paper is to describe the role of Community Engagement in the DCEL model, and examine the benefits of community engagement for learners and communities towards fulfilling NOSM’s social accountability mandate.

## Methods

A variety of studies using different methods provide the evidence on which this paper is based. These studies include research collaborations between NOSM and the Centre for Rural and Northern Health Research (CRaNHR), which track NOSM undergraduate and postgraduate medical learners,^22^ as well as assessing the socio-economic benefits of NOSM.^23^ Administrative data from NOSM and external sources, as well as faculty led surveys and interviews of students, graduates, and other key informants provided the data for these studies. In addition to the tracking and impact studies, there have been specific research studies focused on the annual Health Sciences Summer Camps, the NOSM MD admissions process, the first year Indigenous Community Experience, the Comprehensive Community Clerkship and the impact of NOSM on physician recruitment in Northern Ontario communities. Ethics approval for these studies was granted by the Research Ethics Boards of Laurentian and Lakehead Universities.

## Results

The results examine the role of community engagement, focusing on the stages of preadmission, admission, and undergraduate learning.

### Pre-Admission community engagement

#### High school camps

The NOSM Health Sciences Summer Camps began in July 2006 with 11 high school students attending the weeklong camp at Lakehead University. Since 2007, there have been week-long camps at both Lakehead and Laurentian Universities every July. A total of 459 high school students from 70 different Northern Ontario communities including Sudbury and Thunder Bay, as well as two students from Iqaluit have attended the camps. Amongst the students were 143 Francophones, 126 Indigenous people, and 20 individuals who are both Indigenous and Francophone. Noting that there is a minimum six-year lag time between the camp and admission to medical school, some high school students from the early years have pursued postsecondary education in health programs including three who became medical students at NOSM. The influence of the Health Science Summer Camp has extended to involve 10 Northern Ontario university students per year who work as volunteers with NOSM medical students as team leads at camps. This allows the volunteers to be mentored by the NOSM medical students, as well as have an opportunity to mentor campers.

### Community engagement and admissions

#### MD admissions

Guided by its social accountability mandate, NOSM seeks to reflect the population distribution of Northern Ontario in each MD program class. The admissions process specifically promotes applicants from Northern Ontario, or similar rural/remote, Indigenous, or Francophone backgrounds (“social accountability groups”). There are no prerequisite courses, however there is a preference for a balanced academic background with some science/mathematics and some arts/humanities courses. The Medical College Admissions Test (MCAT) is not used because it has never been validated for Indigenous or Francophone people. Also, there are concerns about a training effect whereby MCAT scores are higher after a MCAT preparatory course.^24^ Applicants from Northern Ontario are likely to be precluded both financially and geographically from accessing MCAT courses.

All applicants with a grade point average (GPA) of 3.0 or above on a 4.0 scale are considered. Each application is scored by two independent raters and applicants are also given a context score that is highest for applicants from Northern Ontario and other targeted backgrounds (“social accountability groups”). Based on the combination of GPA, application score and context score, the top 320 applicants (400 prior to 2013) are invited for interviews. NOSM uses Multiple Mini Interviews (MMI) developed originally by McMaster University consisting of 10 one-question interviews developed by NOSM to be relevant to the Northern Ontario context****. Meaningful community engagement is built into the admission process as each year, up to 128 community, faculty, and student volunteers undertake approximately 4000 assessments of written applications to derive the application score. In addition, each year over 100 volunteers serve as interviewers, of whom approximately one third are faculty members, one third are learners, and one third are community members who, in 2016, were drawn from 39 communities.

Between 2006 and 2015, there were 17,358 qualified applicants for the NOSM MD program of whom 3,705 (21.3%) participated in interviews, and 610 (3.5%) were admitted into the program. Northern (defined as Northern Ontario and the Canadian Territories) and Indigenous applicants have the highest admission rates (18% and 11%, respectively). Admission rates for Francophone and rural/remote applicants (7% and 6%, respectively) are double that for all applicants (3.5%). In assessing how each group moves from the applicant pool to the interview stage, all social accountability groups are selected for interviews at rates that are 2 to 4 times higher than other applicants. For instance, 80% of Northern background applicants are invited to an interview compared with 19% of non-northern applicants. Percentages are very similar for Indigenous applicants (80%) versus non-Indigenous applicants (18%). In progressing from interviews to offers of admission, Northern and Indigenous applicants are offered admission at rates that are 1.5 and 3 times higher than those of their Non-Northern and Non-Indigenous peers, respectively.

At the last stage, moving from an offer of admission to accepting the offer, Northern background applicants accept these offers at 1.6 times the rate of their Non-Northern background peers. In comparison, proportionally fewer Indigenous applicants accept the offer than Non-Indigenous applicants (51% versus 82%). In particular, Indigenous applicants from non-Northern regions have low acceptance rates: they accept 30% of admission offers compared with 72% for Indigenous applicants from Northern regions. Follow-up of Indigenous applicants who have not accepted admission offers at NOSM revealed that they have accepted admission offers at other medical schools, usually closer to their home, and/or in some cases accepted an offer from McMaster University because of the three-year MD program (compared with NOSM’s four-year program).

### Community engagement in curriculum delivery

#### Indigenous cultural immersion

Students are placed in pairs in one of 36 Indigenous communities during the final module of their first year. The placement is intended to support students in the development of culturally safe care for Indigenous patients. The placement provides an opportunity for students to begin developing community relationships as they explore the context of Indigenous health care in Northern Ontario.^14^ A faculty led mixed methods study of the impact of the immersion experience on NOSM students and graduates included semi-structured interviews involving eight NOSM MD graduates from 2009 and 2010 who undertook their Indigenous placements in First Nations, including one remote fly-in community. Five graduates are now family physicians and all have Indigenous patients. Key themes arising from the interviews all centred around the common thread of the value of community-engaged learning. “…we were with a nurse practitioner for some of the placement… Seeing how she kind of dealt with more culturally sensitive issues… and incorporated traditional medicine was probably the take home message that I got from that.*”* (Participant 1). These sentiments are echoed by a community member who wrote in a recent commentary:^25^ “We get to teach our values, our customs, and our ways in a practical and hands-on manner.”

Many of the graduates’ comments included references to greater understanding and empathy with patients from the cultural groups of Northern Ontario, “I think that you gain… insight from this experience, how culture does play a huge part in daily life and decisions then that is true of all culture. That is true of all races.” (Participant 4); “All of those things that we would consider basically standard but are not there in some of these smaller fly-in reserves.” (Participant 7); and “Everybody needs to be treated the same way if you’re from any country in the world, any culture in the world you need to be treated the same way… just treat each other as humans.” (Participant 8).

One Indigenous participant, a NOSM graduate, had lived experience and knowledge of Indigenous culture. For this person, the impact of the community-engaged curriculum was, at the time, on fellow students and the relationship building between NOSM and Indigenous communities. “It’s hard for me to separate because I’m an Aboriginal person… So I mean, this one experience for me may not have prepared me for practice but what it did do was help me see how the medical school is impacting others. How it’s that link between the Aboriginal community and the school is so important and how my colleagues are changed. So it makes me feel very positive about the experience because of that.” (Participant 4).

As a final requirement of the placement students prepare a reflective presentation that is shared first with their host community and then, upon return to the university, with classmates and faculty. Students viewed this activity as worthwhile in both community and university contexts. “I find for the members of the community… I felt it was more important to them. We had a presentation at the end in terms of what we’ve done, what we’ve learnt. And I think, don’t know, half the community was there. Like, for me I felt like they got a lot from it.” (Participant 3).

Analysis of these interviews highlighted the need to ensure that communities are full partners in the process. Most former students reported feeling welcomed and integrated into community life. “We were completely invited into the community… We had more than one celebration with the drummers that came out and the dancing and the feast and we were absolutely adopted by that community.” (Participant 5). However, when in one community where the placement coordination responsibilities fell to non-local nurses, then the experience was different: “I wouldn’t characterize it as welcoming necessarily. The two nurses who worked at the nursing station there were reasonably welcoming. … But we didn’t really get too much of a reception from members of the community at large” (Participant 7). Although there were noted exceptions the overall evaluation from the respondents was that the cultural immersion experience was worthwhile, had lasting effects on their knowledge and practice, and provided an effective learning environment.

#### Comprehensive Community Clerkship (CCC)

After the first CCC, interviews were conducted in 2008 with 46 people, including students, faculty members, site liaison clinicians, other clinical faculty members, site administrative co-ordinators, and health service managers.^26^ Respondents were overwhelmingly positive about the CCC. Students experienced a great variety of exposure to a wide range of clinical problems, and, through taking responsibility for patient care, they reported having developed clinical competence as well as in overall maturity. Students were actively engaged in their learning process, and sites were able to offer variety and flexibility. Students were exposed to rural family practice and primary care, to the health care team and to the roles of a doctor in rural and remote communities. The experience of continuity of care of patients, and ongoing, longitudinal relationships both with patients and with colleagues, enhanced this exposure and learning. The impact of continuity of care alone on students is a major benefit; students see and learn about the whole life cycle of their patients, their health and disease, rather than learning around the acute events and, often, highly complex diseases that characterize tertiary care hospitals. Students reported that their learning was further enhanced by the mentoring relationships that developed between preceptors and students. Preceptors in turn reported being stimulated by the experience, as were members of the broader health care team. In addition, preceptors and health service workers appreciated the longitudinal relationships with students, the extra assistance students provided in patient care and the value of faculty development. The potential for the CCC as a recruitment and retention tool was seen to be important.

In 2011-12, the lived experiences of 12 CCC students were explored using “guided walk” interviews before, during and after their clerkship year.^27,28^ This specific methodological approach was suggested by recent NOSM graduates who were key informants to the study. Findings from this research highlighted adaptive strategies involved in the transition processes of clerkship students: from classroom to clinical learning; dealing with disorientation and restoring balance; and forming their professional identity as a future physician. This research also identified students’ sources of social support during the CCC, specifically preceptors, peers, family, health professionals and community members. The contribution of general community members stands out as a feature of the CCC. The students, without exception, described their reception as welcoming and also commented on how the communities demonstrated an interest in their achievements. The students’ integration was largely facilitated by support from members of the community in promoting broadly that the medical students were completing their clinical training there, and that together the community collectively contributed to providing a positive learning experience. The students felt the patients they encountered in clinical settings had increased awareness of their presence in the community which facilitated the patients’ willingness to allow the students to participate in their care. One student commented that “We do feel as though the community enjoys having us here. That in itself is supportive. Not once have I felt that we aren’t really accepted [or] that they don’t want the medical students here.”

Entry and exit interviews of NOSM MD students and subsequent interviews of practising NOSM graduates have provided further data on the student experience, including their perceptions of career directions and choice of practice location, and their experience of rural generalism.^29^ A NOSM student focus group undertaken by the Canadian Federation of Medical Students (CFMS) in 2010 indicated that: “clinical experiences during (the CCC) are more substantial than anything in traditional medical school experience” and that the CCC: “creates ‘generalists’ and encourages students to maintain a broad focus.” NOSM students and graduates consider generalist care as a comprehensive service with a strong focus on responding to the health needs of the community they serve, reflecting adherence to social accountability. A rural medicine “true generalist” is viewed as a physician who provides care ranging from promoting prevention to performing specialist tasks. One student observed that: “Having had eight months in [my CCC community] last year, I quite enjoyed it and I see that that would be a place I would be happy practicing in. The bottom line is that one can always learn. The key of rural practice is to stay resourceful and learning all the time.” In contemplating the practical, embedded CCC rural practice experience, another student reflecting on rural medicine commented that: “You don’t know it until you live it.”

## Discussion

It has been twelve years since the official opening of the School in 2005, and NOSM is recognised for its success in fulfilling its social accountability mandate. Ninety-two percent of all NOSM students come from Northern Ontario with substantial inclusion of Indigenous (7%) and Francophone (22%) students. Most years all graduating students have been matched to residency programs in the first round of the Canadian Resident Matching Service (CaRMS) with 62% of NOSM graduates having matched to family practice (predominantly rural) training. Seventy percent of residents training at NOSM (graduates from both NOSM and from other medical schools) have chosen to practice in Northern Ontario after completing their training (including 22% choosing small rural communities); and 94% of the doctors who completed undergraduate and postgraduate education with NOSM are practising in Northern Ontario, including 33% in remote rural communities.^5,30^ Many NOSM graduates are now faculty members and an increasing numbers have taken on academic leadership roles in the School.

Findings from various studies highlight community engagement during all stages of the physician’s career path as central to NOSM’s distributed learning model. Active community participation is a feature of: the NOSM week-long Health Sciences Summer Camps having drawn high school students from 70 Northern Ontario communities; the admissions process to the NOSM MD program each year including up to 128 volunteers assessing written applications and over 100 volunteer interviewers; the four-week Indigenous Integrated Community Experience for all NOSM first year students involving 36 Indigenous communities; and the eight-month Comprehensive Community Clerkship (CCC) during which all students live in one of 15 communities and learn their core clinical medicine from the community, family practice perspective. In general, both learners and communities report net benefits from participation in NOSM programs.

The term community engagement has been adopted by universities to describe relationships which vary from student recruitment to fundraising to formal partnerships which achieve specific purposes. Generally, universities are in large population centres so community engagement is particularly challenging for remote rural communities.^31^ For NOSM, community engagement occurs through interdependent partnerships between the School and the communities.^10,11^ This community engagement for mutual benefit is, in reality, quite challenging. In general, communities view academic institutions as distant “ivory towers” that only approach communities when they are looking to benefit the institution. This preconception has been the case particularly for Indigenous communities. Consequently, engaging communities as genuine contributors and as shared decision makers requires considerable effort. Effective strategies that promote mutually beneficial partnerships involve frequent discussions and regular face-to-face contact between NOSM personnel and community members. This relationship is facilitated by formal collaboration agreements and organizational structures such as the 17 Local NOSM Groups that function as steering committees in communities and are a mechanism by which NOSM is a part of the community and the community is a part of the School. In addition, there are two Reference Groups that are advisory committees on the health needs of Indigenous and Francophone populations in Northern Ontario and staff in the communities are hired to work for the School (e.g., Regional Indigenous Coordinators for the Indigenous community experience). It is an ongoing challenge to facilitate meaningful, reciprocal collaboration and ensure that suggested improvements are made, while meeting accreditation criteria and other external constraints.

There is evidence that NOSM’s community engagement approach is beneficial to the communities. For example, researchers from the University of Toronto and Waasegiizhig Nanaandawe’iyewigamig Health Access Centre who conducted a recent study of medical learner elective attachments in Kenora, Ontario, found a difference between trainees from NOSM and from other schools in Ontario.^31^ Interviewees observed that NOSM learners had superior baseline knowledge on the historical, political and geographical issues affecting rural communities, including Indigenous communities, and a sound understanding of the social deprivation that exists in some First Nation communities. In addition, a joint CRaNHR-NOSM study found that NOSM is making a substantial economic contribution to the communities of Northern Ontario not only directly in terms of new economic activity but also indirectly through economic opportunities that are incidental to specific NOSM activities.^32^ Another CRaNHR-NOSM study showed that small communities that had previously struggled to recruit and retain physicians have moved from perpetual crisis mode to planning ahead.^33^ Before NOSM, the eight rural communities in this study had approximately 30 full-time equivalent (FTE) physician vacancies, now they have but one vacant FTE physician position. On the other hand, not all Northern Ontario communities have benefited, including a few communities in this study, and five communities in another study conducted on behalf of NOSM. Both NOSM and the partner communities need to work closely together with adaptability, visibility, continuity, reciprocity and humility.^34^

A key element of the NOSM model is supporting local physicians and other health care providers in communities as the principal teachers of medical students. The vast majority of NOSM’s 1400 faculty members are stipendiary clinical faculty practising in the distributed communities of the School’s campus, Northern Ontario. The NOSM Faculty appointment and promotion policy recognises all faculty members as of equal standing with the same opportunity for academic promotion and career progression. This provides particular challenges for faculty development that are addressed through: 1) distributed faculty development: an annual faculty development conference, Northern Constellations, which brings together over 250 faculty members for two days of intensive interactive workshop sessions; and 2) locally: Local Education Groups established by the NOSM academic alternative funding plan, overseen by the Northern Ontario Academic Medicine Association (NOAMA).^35^

In general, the findings reported in this article are consistent with the review of Community Engaged Medical Education literature that found benefits for learners, for communities and for the academic institutions.^36^ However, there are many challenges still to be addressed, particularly supporting distributed faculty members to pursue academic careers while staying in remote rural communities and working with First Nations to improve healthcare and training in remote First Nation communities. The NOSM Continuing Education and Professional Development (CEPD) program has begun an initiative entitled “Assessing Perceived and Unperceived Needs of Northern Ontario Health Care Professionals for CanMEDS Competencies beyond that of Medical Expert” with the goal of understanding the educational needs of Northern Ontario clinical faculty and to develop CME strategies to meet their needs. Also, NOSM is in the process of establishing a Master Medical Studies program, which will use the DCEL model to encourage distributed clinical faculty members to undertake research and other academic activities while staying in their community and clinical settings with likely added value to the communities. In addition, NOSM is in the process of establishing a Remote First Nations Health stream in its family medicine residency program in response to the health care needs articulated by First Nations communities.

The research presented in this paper focuses primarily on undergraduate medical education at one medical school, the Northern Ontario School of Medicine with no external comparators and no consideration of other possible contributors to NOSM’s social accountability mandate. Further research is required to explore the experiences of communities in-depth, particularly those that currently have less direct involvement with NOSM, as well as studies of NOSM’s community engagement in postgraduate medical education, broader health workforce education and research. There is potential for comparative studies with other schools in Canada, as well as in other parts of the world. For instance, NOSM is a member of the Training for Health Equity network (THEnet)^37^ and NOSM-CRaNHR researchers are participating in studies that explore implementation, evaluation and outcomes of these socially accountable schools. Within Northern Ontario, further research will examine practice characteristics of NOSM graduates including studies that will examine the range of services provided, as well as patient populations served by these graduates.

### Conclusion

This paper has described the role of Community Engagement in the DCEL model, and explored the benefits for learners and communities of community engagement. Community members’ active participation in preadmission activities, the MD program admissions process, and in enhancing medical students’ and residents’ education and training experiences all contribute to NOSM’s success in fulfilling its social accountability mandate.
